# Severe fracture-dislocation of the thoracic spine without any neurological deficit

**DOI:** 10.1186/s12957-016-1070-7

**Published:** 2017-01-05

**Authors:** Shuai Zhang, Ting-Bin Yan

**Affiliations:** Department of Orthopedic Surgery, Qilu Hospital of Shandong University, No. 107 Wenhuaxi Road, Jinan, Shandong 250012 China

**Keywords:** Complete fracture-dislocation, Thoracic spine, Neurological deficit

## Abstract

**Background:**

Fracture-dislocations of the thoracic spine without spinal cord injury are very rare.

**Case presentation:**

A 35-year-old woman presented to our emergency department with complete T6-7 fracture-dislocation without any neurological loss had undergone a surgical reduction and fixation.

**Conclusions:**

The radiological severity of fracture-dislocation pattern doesn’t correlate sometimes with the clinical manifestation.

## Background

Due to the unique sagittal orientation of facet joints and the presence of the costotransverse articulation of the thoracic spine segment, they are mechanically more stable to axial and horizontal translation [1, 2]. In this region, the spinal canal is narrowed, with less free space between the cord and the osseous ring. The central thoracic spine also has a relatively sparse blood supply [3]. Fracture-dislocation of the thoracic spine, often accompanied with spinal cord injury, is usually caused by high-velocity impact or other high-energy injury. Complete neurological dysfunction may occur in the most severe spinal fracture-dislocation cases. However, case reports of fracture-dislocation of the thoracic spine without paraplegia are scarce in the existing literature [4–14]. Patients with thoracic spinal fracture-dislocation without neurological symptoms and costal fractures are rare [15]. Here, we report a 35-year-old female patient with a complete anteroposterior dislocation of thoracic vertebral (AO-B2.3.1 according to AO classification) without neurological deficit. Moreover, we discuss the clinical and radiological features, injury mechanism, and treatment of the thoracic spinal fracture-dislocation.

## Case presentation

A 35-year-old woman presented to us after an accident in which her back was impacted by an 80-kg-heavy-giant rubber tire with metal wheel hub. When the tire fell down from about 10 m high, the patient was standing right under it and the middle thoracic back was hit by the tire from above. She felt pain in her back and remained in place until emergency personnel arrived. After conservative management in the local hospital for 3 days, the patient was transferred to our hospital safely. During the 3 days in the local hospital, the patient received neurotrophic factors and antithrombotic reagent given by venous transfusion without any reduction treatment and she hesitated to accept a surgery. Upon examination, her blood pressure was 142/94 mmHg, pulse rate 60 per minute, respiratory rate 15 per minute, and body temperature 37 °C. She denied numbness and weakness in her extremities. Physical examination revealed that she was neurologically intact without focal sensory or motor deficits and had normal reflexes. Neurological examinations revealed a Frankel grade E. According to ASIA scoring system, the patient got 112 points for light touch sensation, 112 points for pain sensation, 50 points for the upper limb main muscles, and 50 points for the lower limb main muscles. No perianal sensory was lost. Normal anal contractility and sphincter reflex were observed. A severe tenderness point was found in the middle back. She had Medical Research Council (MRC) grade 5 power in both lower limbs. Associated injuries were pulmonary contusion resulting in right pleural effusion, which was treated by chest tube insertion in the local hospital. No neurogenic bladder or fecal incontinence was observed.

**Fig. 1:**
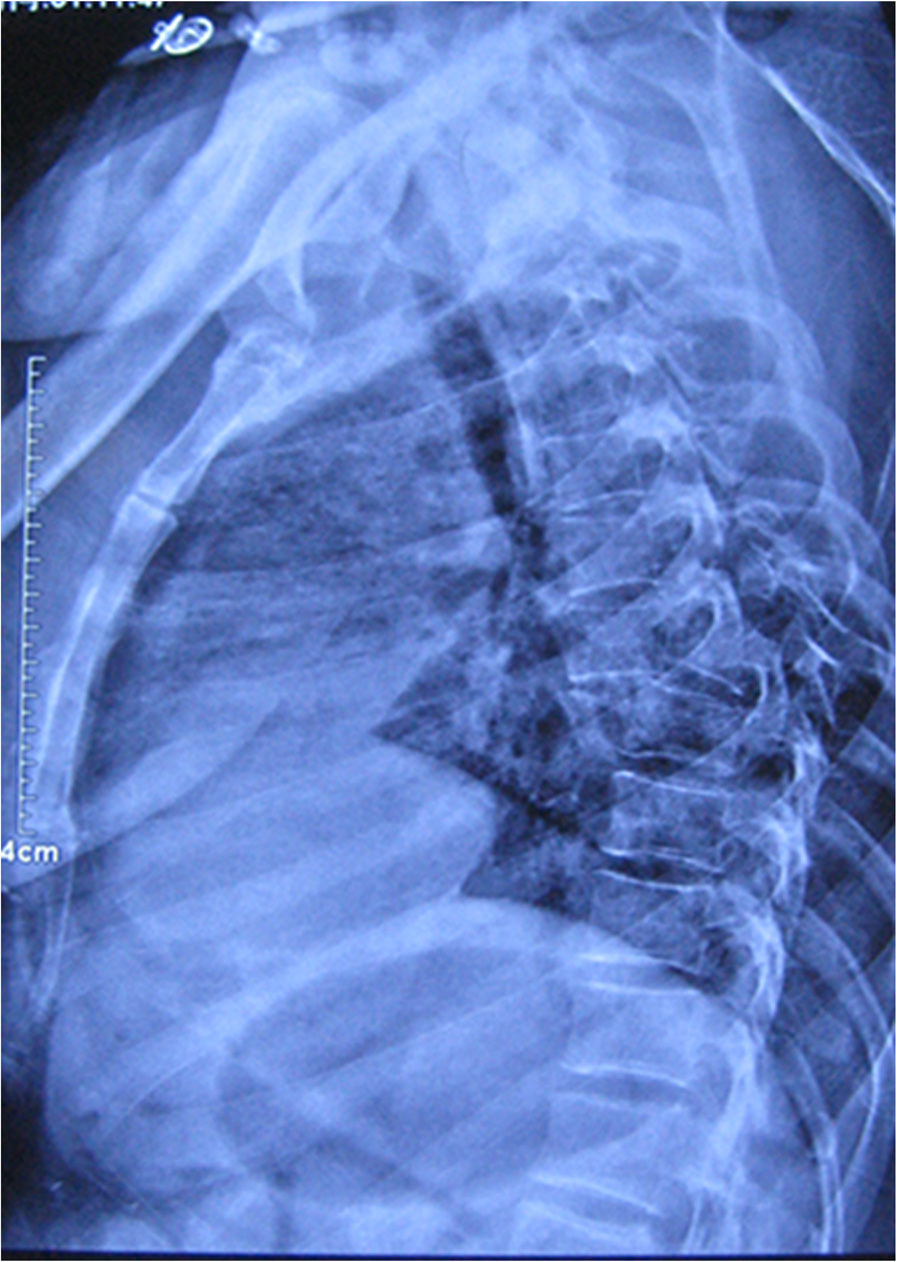
Preoperative lateral radiographs of the thoracic spine revealed fracture and anterior-posterior dislocation of the T6 and T7 vertebrae

**Fig. 2:**
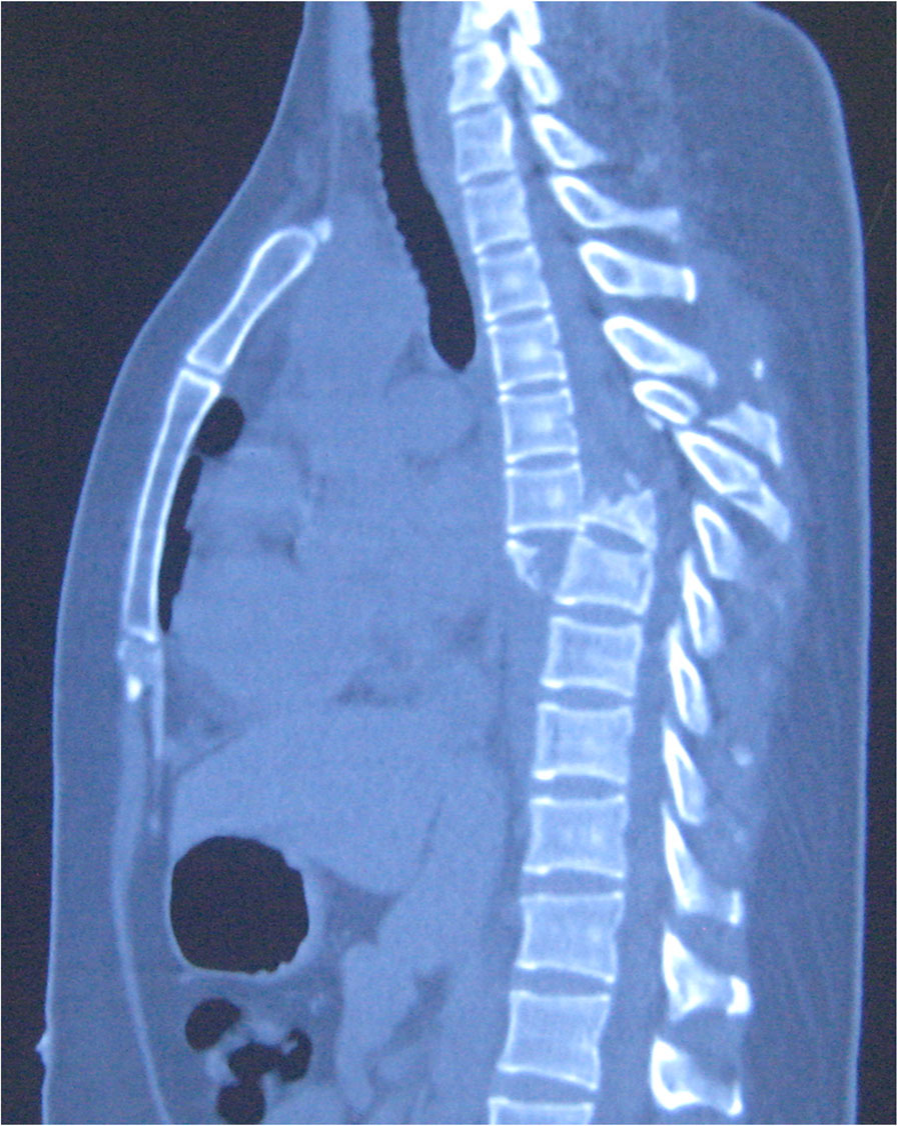
Preoperative sagittal view of computed tomographic scans of the thoracic spine revealed the burst fractures of T7 vertebrae and complete dislocation of T6 vertebrae

**Fig. 3:**
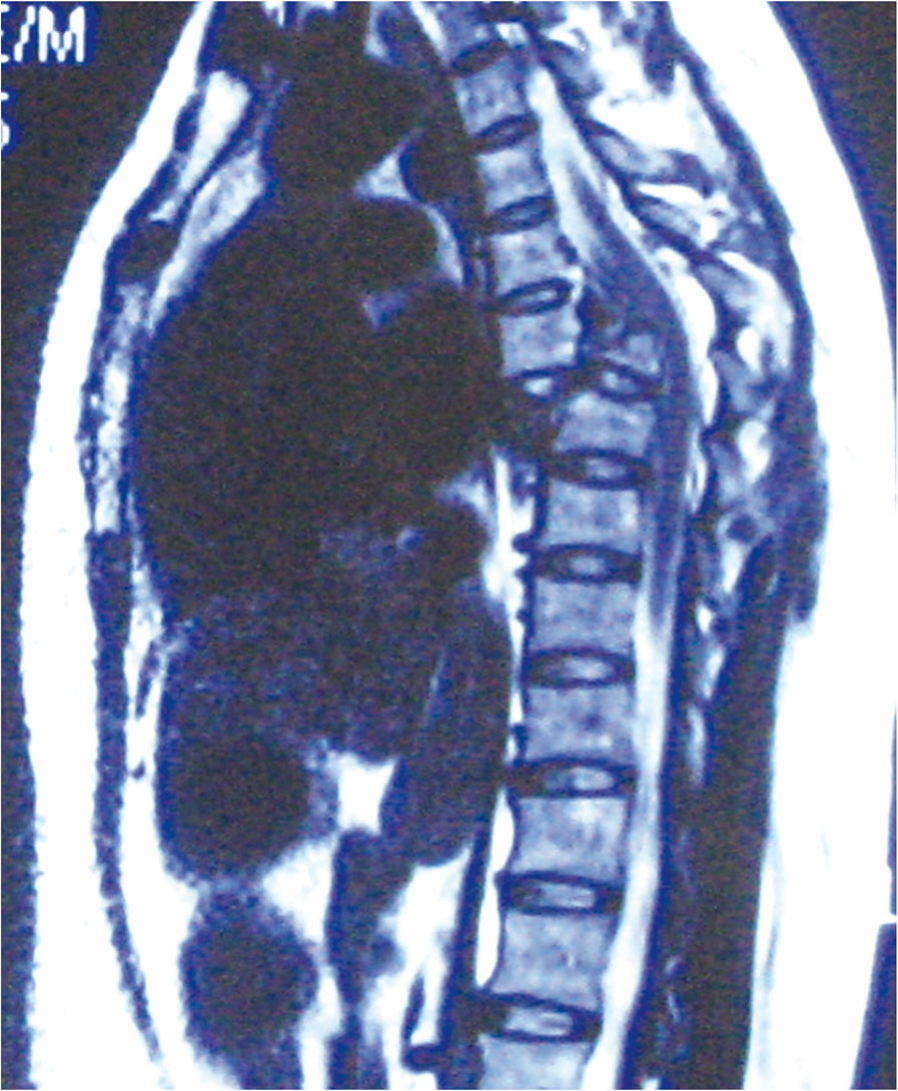
The sagittal MR images revealed that the spinal cord was well decompressed spontaneously

Spine radiography revealed a burst fracture of T7 with complete fracture-dislocation and sagittal displacement of the T6-7 vertebrae (Fig. 1). Spinal computed tomography revealed fractures involving the left pedicle of T4; spinous process, vertebral laminae, and bilateral pedicles of the T5 and T6; spinous process of T7; and both pedicles of the T8 vertebra. The fractures in the bilateral facet joints of the T6/7 vertebrae and T7 body burst fracture resulted in the dislocation between the T6 and T7 vertebrae. There was no retropulsion of bony fragments into the spinal canal, and spinal canal was expanded because of the fracture of pedicles (Fig. 2). Magnetic resonance imaging showed spinal fracture-dislocation of T6 to T7 and no abnormal high signal intensity of T2 weight image (Fig. 3). According to the records of the local hospital, 1.8 g methylprednisolone were administered at the local hospital about 2 h after admission and decompressive immobilized surgery was performed 3 days after arriving in our department.

**Fig. 4:**
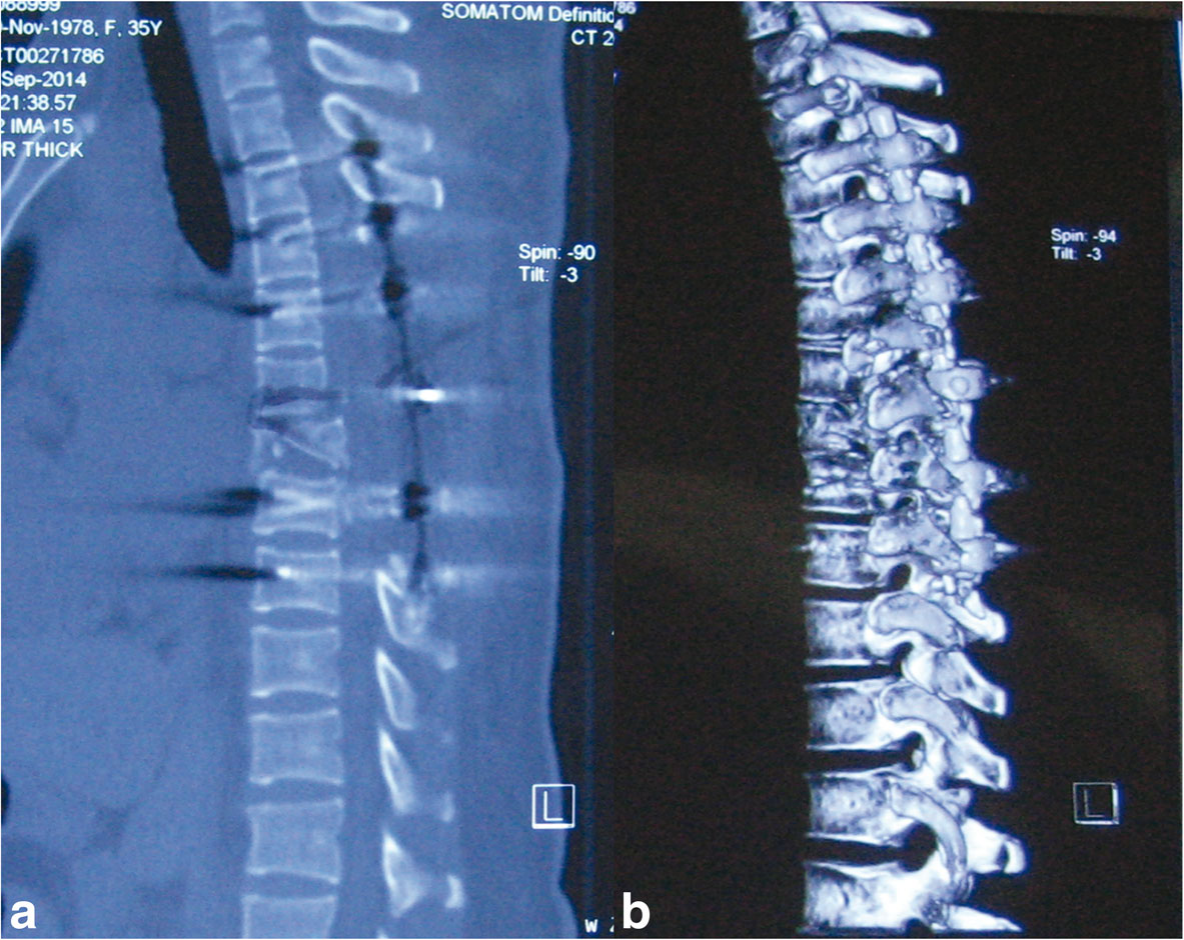
Postoperative computed tomographic scans of the thoracic spine. **a** Axial view revealed the decompression of the spinal canal and reduction of T6&T7 dislocation. **b** The 3-dimensional reconstruction revealed the normal alignment after the insertion of instruments

Under general anesthesia, the patient was carefully placed in a prone position. A traumatic hematoma was evacuated on the fascia after a thoracic midline vertical incision. The erector muscle of the spine from T5 to T8, adjacent part of the latissimus dorsi, and corresponding skin and superficial fascia were found to be damaged severely. The fracture formed a spontaneous decompressive sparing canal containing the spinal cord. The vertebral body of T7 was broken almost vertically and the majority detached posteriorly. The bilateral superior facet of T7 was broken and dislocated behind the inferior facet of T6. No hematoma or dura membrane tear was found. After extensive decompression from T5 to T8 with a high-speed burr, the dura and spinal cord appeared as normal without tearing except for the displacement. Realignment of the spine was achieved with rods after placement of transpedicle screws in the T3, T4, T5, T8, and T9 vertebrae (Fig. 4a, b). During the reduction, two rods were placed in the screws firstly. The nuts of screws in T8 and T9 were tightened while the other nuts were not. A distractor was applied between T4 screw and a powerful plier biting on the proximal of rods. Then, the dislocated vertebrae were replaced with distraction between T4 and pliers. All the nuts were tightened after reduction of vertebrae. Finally, posterolateral bony fusion of T3 through T9 was performed. Intra-operative neurophysiological monitoring was applied throughout the operation. The massive blood loss from comminuted fracture of the vertebral body was solved by autologous blood transfusion.

No deteriorated neurological function was observed postoperatively. The patient was discharged on day 27 and could walk without assistance. At 6-and 12-month follow-up, the patient has recovered well. At 6 months after the injury, the patient returned for reexamination walking on foot without any brace. At the 12-month follow-up, the patient was able to walk 2 km a day continuously and returned to her job.

## Discussion

Fracture-dislocation of the thoracic spine is a common result of high-velocity motor accident or crash injuries. Such spinal injury is the most unstable type with failure of all three columns [11]. Dural tear and paraplegia often accompany such injuries. Neural injury is caused by bone fragments and/or encroachment of the spinal canal due to translational displacement. Classification of fracture-dislocations of the thoracic spine has been accomplished according to different mechanisms [2]. Magerl considered slice fractures to be more dangerous than rotational shear oblique fractures with regard to spinal cord compression because of the shear in the sagittal direction. Thus, case reports of complete fracture-dislocation on the sagittal view of the thoracic spine without paraplegia are rare in the literatures, but ours is just such a case.

The most crucial element to preserve intact neurological function in patients with thoracic spine fracture-dislocation is to decompress the spinal elements spontaneously while sparing the spinal cord. Fracture of the pedicle or facets at involved levels is considered a vital precondition for widening the spinal canal to a significant extent [15].

It is critical for clinical practitioners to diagnose a patient with complete fracture-dislocation of the thoracic spine with no paraplegia or severe neurological deficits, as inappropriate maneuvering of the spine may lead to dangerous impairments of the spinal cord [16]. Appropriate immobilization must be ensured before any investigation is done. Currently, the best diagnostic method for evaluating the severity and stability of the spine is computed tomography with a high quality reconstruction of the spine [17].

Due to the instability of fracture-dislocation, surgical treatment is recommended to realign the spine and prevent secondary injury to the spinal cord [18]. Early surgery has the benefits of the preservation of neurological function, the provision of early mobilization, earlier rehabilitation, shorter hospital stay, and a reduction in associated complications. Operation for reduction of dislocation for such cases, with or without fusion with instruments, has been reported [5–8, 11, 12, 15]. Simpson AH etc. reported 2 cases of thoracic translocation with no neurological deficit and one of them received surgical reduction with Harrington instruments [9]. The single posterior approach was carried out to avoid iatrogenic injury of the spinal cord in hand and to avoid the intractable complications of an anterior approach [19, 20]. Weber SC etc. operated reduction surgery by anterior approach with Harrington rods and fixation with anterior AO/ASIF plate and posterior rods and wires [5]. Reduction and fixation with transpedicular screws and rods by single posterior approach, which is similar to our methods, was reported by Jiang B etc. [15]. Conservative management was also applied to such rare thoracic-dislocation patients according to some literatures [4, 9, 10, 14]. Continuous halo-femoral traction was an optional treatment to reduce the dislocation [4, 14]. The key data of cases of complete thoracic fracture-dislocation with neural sparing, including reduction techniques have been compiled in Table 1.

**Table 1:** Date compiled from previously published cases of thoracic fracture-dislocation with neural sparing

Authors	Level	Dislocation	Cause	Surgery	Reduction technique
Gertzbein SD etc. [4]	T5-6	Lateral	Plane engine failure	No	Halo-femoral traction
Weber SC etc. [5]	T7-8	Lateral	Motorcycle accident	Yes	Harrington distraction rods from anterior
Harryman DT etc. [6]	T6-7	Lateral	Overturn of jeep	Yes	Not mentioned
Sasson A etc. [7]	T9-10	Anterior	Automobile accident	Yes	Harrington distraction rods from posterior
de Lucas JC etc. [8]	T8-9	Lateral and vertical	Automobile accident	Yes	Not mentioned
Simpson AH etc. [9]	T9-10	Anterior-lateral	Traffic accident	Yes	Harrington instrumentation
Simpson AH etc. [9]	T6-7	Lateral	Thrown off a horse	No	Not mentioned
Miyasaka Y etc. [10]	T6-7	Anterior-lateral	Traffic accident	No	Direct traction of both femur
Korovessis P etc. [11]	T5-6	Lateral	Motorcycle crash	Yes	2 Luque L-rods with sublaminar wires from posterior
Potter MJ etc. [12]	T4-5	Anterior	Fell down a single flight of stone steps	Yes	Not mentioned
Anthes TB etc. [13]	T4-6	Anterior-lateral	Motocycle crash	Not mentioned	Not mentioned
Uriarte E etc. [14]	T6-7	Lateral	Motorbike accident	No	Continuous halo-femoral traction
Jiang B etc. [15]	T6-7	Anterior-latera	Motocycle crash	Yes	Transpedicular screws and rods from posterior

## Conclusions

Complete fracture-dislocation of the thoracic spine without paraplegia is rare and treatable. Clinical practitioners should pay more attention to patients diagnosed with spinal trauma without paraplegia, as inappropriate maneuvering of the spine may lead to dangerous impairments of the spinal cord. To treat such an unstable spine, the best management policy is early surgical intervention.

## Abbreviations

ASIA: American Spinal Injury Association
MRC: Medical Research Council
